# Neuromechanical effects and predictive profiling of sprint priming strategies in elite youth sprinters

**DOI:** 10.5114/biolsport.2026.154150

**Published:** 2025-09-12

**Authors:** Krzysztof Kotuła, Aleksander Matusiński, Adam Maszczyk, Lucas A. Pereira, Adam Zajac, Irineu Loturco

**Affiliations:** 1 Institute of Sport Sciences, Jerzy Kukuczka Academy of Physical Education, Katowice, Poland; 2 NAR – Nucleus of High Performance in Sport, São Paulo, Brazil; 3 Department of Human Movement Sciences, Federal University of São Paulo, UNIFESP, São Paulo, Brazil; 4 UCAM Research Center for High Performance Sport, UCAM – Universidad Católica de Murcia, Murcia, Spain; 5 Facultad de Deporte, UCAM – Universidad Católica de Murcia, Murcia, Spain

**Keywords:** Sprint velocity, Athletic performance, Track and field, Resisted sprinting, Priming strategies

## Abstract

This study aimed to compare the acute biomechanical effects of three distinct sprint-specific priming strategies – resisted sprinting, assisted sprinting (i.e., overspeed), and technical wicket drills – on neuromechanical performance during 50-m sprint trials in elite youth sprinters. Twelve nationally ranked female youth sprinters (17.3 ± 0.8 years) participated in a randomized, repeated-measures protocol. Each athlete performed baseline 50-m maximal sprints, followed by three separate priming interventions, with performance re-evaluated at 24 h and 48 h post-activation. Key outcome measures included 50-m sprint time, reactive strength index (RSI), ground contact time (GCT), flight time (FT), step length, step frequency, duty factor, and asymmetry metrics. Data were analyzed using repeated-measures ANOVA, principal component analysis (PCA), k-means clustering, and machine learning classifiers. Assisted sprinting produced the greatest improvements in RSI (+0.13) and the largest reductions in GCT (−16 ms) at 48 h post-activation (*p* < 0.001). Resisted sprinting significantly increased step length (+0.09 m), while technical drills improved interlimb asymmetry and mediolateral control. PCA revealed two primary adaptation domains: PC1 (RSI, GCT, FT) and PC2 (interlimb asymmetry, mediolateral sway, and step frequency). Machine learning models (AUC = 0.83–0.85) identified the priming strategy, baseline asymmetry, and step frequency as the strongest predictors of ≥ 10% improvement in RSI. Sprint priming strategies elicited distinct neuromechanical responses that can be assessed during 50-m sprint trials. The overspeed protocol most effectively enhances force-time capacity and sprint performance, whereas technical drills primarily improve coordination. Integrating multivariate modeling facilitates the individualized prescription of priming protocols, offering a flexible and evidence-based approach to sprint optimization and athlete development.

## INTRODUCTION

Maximizing sprint performance is a critical objective in sport science and athletic preparation. The complex interplay between neuromuscular readiness, biomechanical efficiency, and power output is essential for optimizing both acceleration and maximal sprint speed [1]. In recent years, growing attention has been given to short- or mid-term activation interventions designed to enhance sprint performance immediately or within 24–48 h following their application [2]. In brief, these strategies involve specific exercises performed prior to competitions or training sessions with the aim of improving subsequent speedrelated abilities and other performance parameters [3]. The physiological basis of these pre-activation activities is multifactorial, involving neural, muscular, metabolic, and hormonal mechanisms and responses [2].

Among these acute strategies, post-activation performance enhancement (PAPE) has emerged as a promising strategy. PAPE protocols typically involve resistance-based or high-intensity conditioning activities designed to transiently potentiate the neuromuscular system [2, 4, 5]. Unlike post-activation potentiation (PAP), which is usually assessed within seconds to a few minutes after the stimulus and is often confounded by fatigue [6, 7], PAPE is believed to result from longer-lasting physiological changes, generally occurring 4–12 min after the stimulus [2, 4, 5]. These changes include enhanced neural function, greater recruitment of type II motor units, alterations in muscle architecture, increased muscle temperature, elevated calcium sensitivity, and higher muscle water content [4, 6].

Several studies have demonstrated that PAPE can improve sprint kinematics, including reductions in ground contact time (GCT), and increases in step length and in the reactive strength index (RSI) across various athletic populations [8–10]. However, the efficacy of different PAPE modalities in top-level athletes (e.g., elite sprinters) remains equivocal. Resisted sprinting, assisted sprinting, and technical drills are commonly used in practice, yet their comparative acute effects on specific biomechanical markers remain largely unexplored and rarely compared [9, 11, 12]. Notably, a systematic review with meta-analysis of PAPE protocols concluded that different types of conditioning activities appear to be ineffective at enhancing sprint speed in competitive sprinters, supporting earlier research indicating that sprinting is a highly stable physical capacity, particularly among elite-level athletes [13–19].

More recently, a new strategy – performed several hours or even one or more days before training or competition – has demonstrated significant effectiveness in enhancing speed and power qualities, even in professional athletes [20–23]. This strategy, known as “priming activities,” has been receiving increasing attention from the sport science community due to its positive effects on performance and its practicality [20, 23]. For example, Cook et al. [24] demonstrated that a priming session consisting of unresisted sprint drills in rugby union players improved 40-m sprint performance 6 h after the protocol. Additionally, Kotuła et al. [25] examined the effects of a session involving assisted sprints at 105% of maximal sprint speed in female sprinters and observed a significant improvement in 30- and 50-m sprint performance 48 h after the priming activity. Lastly, Pino-Mulero et al. [26] reported meaningful increases in 20-m sprinting speed 24 h after a priming session in which semi-professional soccer players performed sled pushes with a load equivalent to 100% of their body mass.

Despite these promising results, the biomechanical and neuromechanical mechanisms underlying the effects of priming protocols on performance variables have not been fully elucidated. Moreover, little is known about inter-individual variability in response to these interventions, especially among youth athletes whose neuromuscular systems are still maturing [27]. The ability to precisely evaluate biomechanical responses using objective metrics – such as RSI, GCT, and asymmetric indices – is crucial for assessing the efficacy of sprintbased priming exercises [28–30]. These data not only provide insights into performance outcomes but also support the identification of individualized responsiveness patterns to specific priming stimuli.

Therefore, the present study aimed to compare the acute responses of a comprehensive set of mechanical variables (e.g., RSI, flight time [FT], GCT, step length) over sprint distances of 10–50 m following three distinct priming strategies in a cohort of elite youth female sprinters. Using a within-subject design and multivariate analysis techniques, this study seeks to elucidate both the overall efficacy and individual variability in response to resisted sprinting, assisted sprinting, and technical activation-based priming protocols (i.e., mini-hurdle wicket drills). All biomechanical and neuromechanical measures were assessed during 50-m maximal sprint trials, encompassing both the acceleration and top-speed phases of sprint running.

## MATERIALS AND METHODS

### Subjects

Twelve elite female youth sprinters (age: 17.3 ± 0.8 years; height: 172.5 ± 5.4 cm; body mass: 54.1 ± 6.2 kg) participated in the study. All athletes were members of the Polish national team and were assessed during the pre-competitive phase of the indoor season. The sprinters were primarily 100-m specialists who also competed in the 60-m (indoor) and, occasionally, the 200-m events. Each participant had at least three years of training experience and had been injuryfree for the preceding three months. Written informed consent was obtained from all athletes and their legal guardians. The study was approved by the Institutional Ethics Committee of the Jerzy Kukuczka Academy of Physical Education in Katowice (approval code: 3/2021 [17.06.2021]).

### Design

A repeated-measures, within-subject design was employed to compare three priming strategies: resisted sprinting, assisted sprinting (i.e., overspeed), and technical drills using wickets. For each intervention, biomechanical assessments were conducted at three standardized time points: baseline (pre-activation), 24 h, and 48 h postactivation, resulting in a total of nine sprint testing sessions per participant. Each testing session was separated by at least one week to ensure adequate recovery and eliminate potential carryover effects. All sessions were conducted under standardized environmental and nutritional conditions to ensure comparability.

### Priming Interventions and 50 m Sprint Testing Protocol

The experimental session was conducted on an indoor-certified synthetic track at the National Olympic Training Center. Prior to the tests, athletes completed a standardized warm-up, which included two 40-m accelerations at 85–90% of maximal effort. Two baseline 50-m all-out sprints were performed, and the better result was taken for further analysis. Similarly, 24 h and 48 h after each form of activation, an additional 2 × 50-m sprints were carried out to determine the priming effects. In all testing sessions and priming protocols, a full recovery period of 5–6 minutes was provided between consecutive sprint trials to allow for complete physiological and neuromuscular recovery. Ten Witty photocell cameras and an Optojump Next-Microgate measurement system (Optojump, Bolzano, Italy) were used to record kinematic sprint variables. The photocells were positioned on the track at the starting line and at the 10-, 20-, 30-, and 50-m marks. Athletes started from a semi-crouch position, aiming to accelerate as fast as possible and reach maximal running speed.

The spatiotemporal properties of the running steps – such as step length, step frequency, GCT, and flight duration – were measured using the Optojump system, comprising two parallel bars (100 cm × 4 cm × 3 cm) that emit and receive invisible LED light beams with a grid resolution of 1 cm. Each bar contains 32 photocells (RX and TX), spaced 4 cm apart and positioned 0.2 cm above the surface. Each footstep interrupts the beams and is detected with 1 ms accuracy. The system operates contact-free and records at a 1,000 Hz sampling rate. For data processing and storage, the device was integrated with a computer. This segmented timing setup allowed for precise temporal resolution of sprint phases across the entire 50-m distance, enabling a detailed spatiotemporal analysis of acceleration patterns and sprint speed profiles. The 50-m sprint distance was selected to capture both the acceleration phase and the transition into top speed, making it a sensitive and representative test for detecting priming-related responses.

For the resisted and assisted (overspeed) sprint activation protocols, a Sprint 1080 motorized resistance and assistance system (1080 Motion AB, Stockholm, Sweden) was used. This system allows precise selection of loads and variables, thus facilitating adaptation to sports training diagnostics and performance assessment. It records running time with an accuracy of 0.01 s, as well as average and peak values for mechanical variables such as force (N), power output (W), and running speed (m/s). The resisted sprint protocol consisted of five 30-m sprints with a resistance equal to 10% of the athletes’ body mass. The overspeed protocol involved five 40-m sprints performed at 105–110% of the maximal sprint speed achieved by each sprinter. The technical protocol comprised five 40-m accelerations over uniformly spaced mini-hurdle wickets, with the spacing adapted to each athlete’s stride length. All interventions were evaluated using repeated 50-m sprint tests, enabling consistent assessment of priming effects on performance-relevant sprint mechanics across comparable distances. The 50-m sprint served as a standardized benchmark for inter-condition comparisons of key neuromechanical variables, including RSI, GCT, and stride-related parameters.

### Data Collection and Processing

Raw data were extracted from XML files and processed using a custom Python pipeline. Neuromechanical and spatiotemporal variables were measured and analyzed as average values across all steps over the 50-m sprint distance. For each condition, key performance indicators were derived from repeated sprint trials and exported to a centralized dataset. The primary neuromechanical outcomes included GCT, FT, step length, step frequency, and RSI. RSI was calculated as the ratio between FT and GCT. Duty factor, defined as the percentage of the stride spent in contact with the ground, was also calculated. Asymmetric indices were derived by comparing bilateral time and distance variables, while body sway metrics in the anteroposterior (AP) and mediolateral (ML) directions were extracted to assess postural control during sprinting. Stride and contact variables were normalized to body height and body mass and subsequently computed to systematically account for inter-individual anthropometric variability. Each of these metrics was treated as a dependent outcome to evaluate the influence of the priming strategy. Data from all five conditions were aligned by athlete identifier and structured into a longformat data frame suitable for repeated-measures statistical modeling. The variables were z-score normalized for principal component analysis (PCA) and machine learning analyses. All measures were categorized into five conceptual domains: anthropometric (age, sex, body mass, and height), spatiotemporal (GCT, FT, duty factor, step length, step frequency, and interlimb asymmetry), stability-related (body sway in AP/ML directions and normalized foot contact), neuromechanical (RSI), and derived metrics (e.g., bilateral asymmetric indices). For clarity, RSI is an efficiency index derived from spatiotemporal timings (FT/GCT), interpreted as a neuromechanical construct, and expressed as a dimensionless measure.

### Statistical Analysis

Descriptive statistics (mean, standard deviation, and 95% confidence intervals) were calculated for each biomechanical variable at all seven time points (i.e., baseline, 24 h, and 48 h post-activation for each of the three priming conditions). A two-way repeated-measures ANOVA (3 conditions × 3 time points: baseline, 24 h, 48 h) was employed to examine the main effects of the priming protocols (resisted, assisted, technical), time (baseline, 24 h, 48 h), and their interaction. Prior to analysis, data were checked for normality and sphericity; when Mauchly’s test indicated a violation of the sphericity assumption, the Greenhouse–Geisser correction was applied. Post hoc comparisons were conducted using Bonferroni-adjusted pairwise tests to identify specific differences between time points within each priming condition and between conditions at matched time points. Cohen’s *d* effect sizes (ES) were also calculated to determine the magnitude of the differences. Bivariate correlations between neuromechanical parameters (i.e., RSI) and spatiotemporal parameters were assessed using Pearson’s product-moment correlation coefficient. All variables met the assumptions of linearity and normality. To explore the underlying data structure and dimensionality, PCA was applied to z-score–standardized spatiotemporal, stability-related, and neuromechanical measures; anthropometric variables and derived metrics were excluded. Components explaining ≥ 80% of the total variance were retained. Based on the principal components, k-means clustering (k = 2) was used to classify athletes as “high” or “moderate” responders. Binary logistic regression models were trained to predict the likelihood of RSI improvement ≥ 10% relative to baseline, using condition, time, and baseline variables (asymmetry, step frequency, and RSI) as predictors. Ensemble machine learning classifiers (Random Forest, XGBoost) were implemented with 5-fold crossvalidation to assess predictive performance and reduce the risk of overfitting. Model discrimination was evaluated via the area under the Receiver Operating Characteristic (ROC) curve (AUC), and feature importance was calculated using Gini impurity (Random Forest) and gain metric (XGBoost). The alpha level was set at *p* < 0.05 for all analyses. All statistical computations were performed using Python (v 3.10) and R (v 4.2.2), utilizing relevant packages including *lme4, scikit-learn, statsmodels*, and *ggplot2*.

## RESULTS

The analysis of sprint times revealed consistent performance improvements (*p* < 0.05) following the different priming strategies at 48 h post-activation across the various distances tested, except for the technical protocol in the 10-m and 20-m sprints (Table 1). Significant interaction effects were observed for RSI (*p* < 0.001), GCT (*p* < 0.01), and step length (*p* = 0.002). RSI significantly increased at both 24 h and 48 h after assisted sprinting compared to the corresponding baseline (*p* = 0.003, ES = 0.84; and *p* = 0.001, ES = 0.91, respectively). In the resisted sprint condition, step length was significantly greater at 24 h post-activation compared to baseline (*p* = 0.017, ES = 0.66). FT significantly increased at 24 h and 48 h post-resisted sprint priming (*p* = 0.029 and *p* = 0.012), while GCT was lowest after assisted sprinting at 48 h (*p* < 0.01 across comparisons). A summary of these time- and condition-dependent changes is presented in Table 2.

**TABLE 1:** Comparison of sprint times among the priming strategies across time points.

Sprint distance	Priming strategy	Base-line	24 h	48 h	p-value	Effect size
**10 m (s)**	Resisted	1.81	1.77	1.75	0.048	0.49
	Supramaximal		1.74	1.72	0.037	0.57
	Technical		1.79	1.78	0.062	0.42

**20 m (s)**	Resisted	3.14	3.09	3.07	0.032	0.54
	Supramaximal		3.05	3.03	0.025	0.63
	Technical		3.12	3.10	0.055	0.46

**30 m (s)**	Resisted	4.36	4.31	4.28	0.021	0.61
	Supramaximal		4.26	4.23	0.017	0.69
	Technical		4.33	4.31	0.049	0.50

**50 m (s)**	Resisted	7.02	6.95	6.90	0.015	0.68
	Supramaximal		6.87	6.83	0.009	0.74
	Technical		6.98	6.96	0.043	0.53

**TABLE 2:** Comparison of biomechanical responses among priming strategies across time points.

Variable	Priming Strategy	Baseline	24 h	48 h	Baseline vs. 24 h p	Baseline vs. 48 h p	Interaction	Baseline × 24 h ES	Baseline × 48 h ES
^*^RSI	**Resisted**	1.85 ± 0.25	1.98 ± 0.25	2.00 ± 0.25	0.045	0.038	p < 0.001	0.51	0.58
	**Supramaximal**		2.06 ± 0.25	2.08 ± 0.25	0.003	0.001		0.84	0.91
	**Technical**		1.97 ± 0.25	1.95 ± 0.25	0.056	0.072		0.47	0.41

**Flight Time (ms)**	**Resisted**	1.15 ± 0.15	1.24 ± 0.15	1.25 ± 0.15	0.029	0.012	p = 0.024	0.62	0.68
	**Supramaximal**		1.23 ± 0.15	1.24 ± 0.15	0.042	0.035		0.54	0.61
	**Technical**		1.21 ± 0.15	1.21 ± 0.15	0.078	0.065		0.38	0.43

**GCT (ms)**	**Resisted**	0.95 ± 0.12	0.88 ± 0.12	0.88 ± 0.12	0.041	0.033	p < 0.01	0.55	0.61
	**Supramaximal**		0.86 ± 0.12	0.85 ± 0.12	0.009	0.008		0.77	0.81
	**Technical**		0.91 ± 0.12	0.91 ± 0.12	0.083	0.091		0.34	0.29

**Step Length (m)**	**Resisted**	2.10 ± 0.18	2.22 ± 0.18	2.22 ± 0.18	0.017	0.021	p = 0.002	0.66	0.64
	**Supramaximal**		2.19 ± 0.18	2.20 ± 0.18	0.048	0.039		0.52	0.57
	**Technical**		2.16 ± 0.18	2.16 ± 0.18	0.095	0.088		0.31	0.33

RSI: reactive strength index; GCT: ground contact time; ES: effect size.

* RSI is a dimensionless measure.

Correlation analysis confirmed internal consistency among forcetime-related variables. RSI was positively correlated with FT (*r* = 0.64, *p* = 0.001) and negatively with GCT (*r* = −0.71, *p* < 0.001) and duty factor (*r* = −0.62, *p* = 0.002), as shown in Table 3. These patterns were consistent across priming modalities but were most pronounced in the overspeed and technical conditions.

**TABLE 3:** Bivariate correlations between RSI and spatiotemporal sprint measures. Correlation coefficients (r) are presented with standard errors (SE), p-values, and direction of associations.

Variable	r	SE	p-value	Direction
Flight Time	0.64	0.10	0.001	Positive
Ground Contact Time	−0.71	0.08	0.001	Negative
Duty Factor	−0.62	0.11	0.002	Negative

**FIG. 1:**
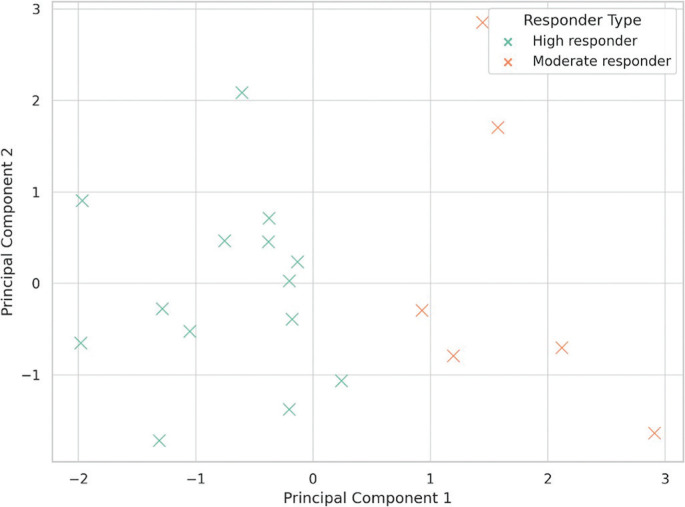
Principal component analysis (PCA) and K-means clustering based on sprint variables. PC1 represents the reactive strength index (RSI), ground contact time (GCT), and flight time (FT); PC2 represents interlimb asymmetry, mediolateral sway, and step frequency. Clustering distinguishing athletes into distinct responder profiles.

PCA revealed two dominant orthogonal components explaining 81.6% of the total variance. PC1 (54.2%) predominantly reflected spatiotemporal timing and neuromechanical efficiency, with high loadings for RSI, FT, and GCT. PC2 (27.4%) represented coordination and stability, with loadings for asymmetry, mediolateral sway, and step frequency. K-means clustering (*k* = 2) identified high responders and moderate responders, with the former group showing pronounced gains in RSI and asymmetry following assisted sprinting and technical priming protocols. The clustering results are visualized in Figure 1, and the component loadings are detailed in Table 4.

**TABLE 4:** Principal component loadings of selected sprint biomechanical variables.

Variable	PC1	PC2
RSI	-0.62	-0.19
GCT	-0.08	0.54
FT	0.47	0.43
Interlimb Asymmetry	0.12	-0.55
Mediolateral Sway	-0.48	0.1
Step Frequency	0.38	-0.43

*Note:* PC1 = RSI, GCT, and FT; PC2 = Interlimb Asymmetry, Mediolateral Sway, and Step Frequency

**FIG. 2:**
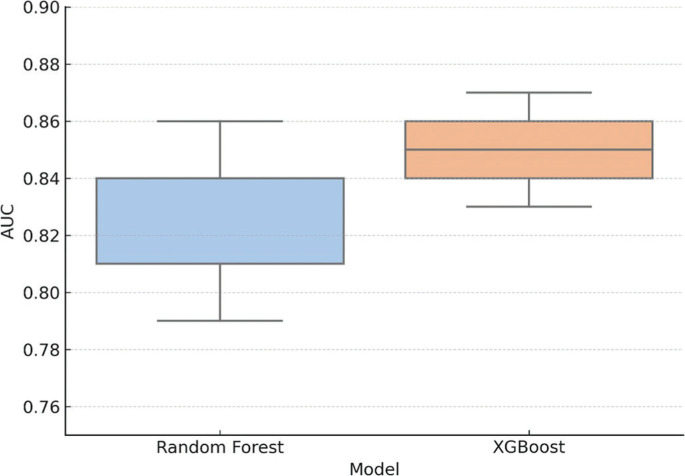
Cross-validated AUC scores for Random Forest and XGBoost classifiers (5-fold cross-validation). AUC = area under the ROC curve. Boxplots show the distribution of AUC values across the five folds for each model, and black dots represent individual fold scores. Both models demonstrated consistently high performance, with XGBoost showing slightly lower fold-wise variance.

To evaluate the predictive potential for RSI improvement, machine learning classifiers were trained using the priming protocol, the time point (24 h or 48 h), baseline asymmetry, and step frequency as predictor variables. Improvement was defined as a ≥ 10% increase relative to each condition’s respective baseline. Both Random Forest and XGBoost classifiers were assessed using stratified 5-fold cross-validation. The AUC was 0.83 (± 0.04) for Random Forest and 0.85 (± 0.03) for XGBoost, indicating strong discriminatory capacity across folds. These findings suggest that model performance was not driven by overfitting or data leakage. AUC values > 0.80 are considered indicative of high predictive accuracy. Fold-specific variability is shown in Figure 2.

**FIG. 3:**
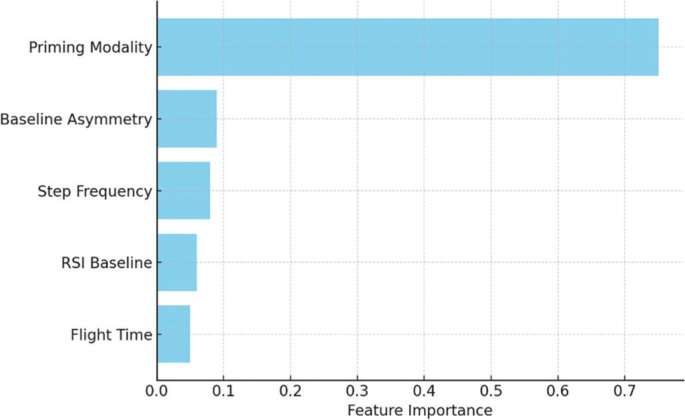
Variable importance in RSI prediction models. Feature importance was derived from the Gini index (Random Forest) and gain metric (XGBoost). Priming strategy and baseline metrics – particularly interlimb asymmetry and step frequency – were the strongest contributors to predictive accuracy.

Logistic regression analysis further identified priming strategy (β = 1.78, OR = 5.93, *p* = 0.015) and baseline asymmetry (β = −0.92, OR = 0.40, *p* = 0.041) as significant predictors of RSI response. Ensemble models consistently rank these features, alongside step frequency, as the most important for outcome prediction. Feature rankings are visualized in Figure 3, and logistic regression coefficients are presented in Table 5.

**TABLE 5:** Logistic regression coefficients for RSI improvement (≥ 10%).

Variable	β	OR	CI_lower	CI_upper
Intercept	-3.61	0.03		
Priming Modality	1.78	5.93	1.47	5.57
Baseline Asymmetry	-0.92	0.40	0.18	0.89
Step Frequency	-0.29	0.75	0.62	0.90

*Note*: β = coefficient; OR = odds ratio; CI = 95% confidence interval. All predictors were z-standardized.

**FIG. 4:**
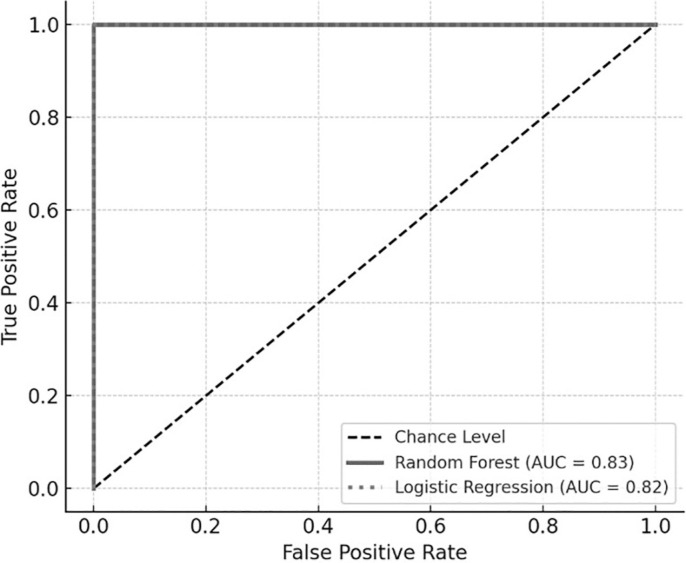
ROC curves for RSI prediction. ROC = receiver operating characteristic. Curves illustrate the classification performance of Random Forest (solid gray line, AUC = 0.83) and logistic regression (dotted gray line, AUC = 0.82). The diagonal dashed line represents chance-level classification.

ROC analysis confirmed the strong classification performance of both predictive models. As shown in Figure 4, the AUC for the Random Forest model was 0.85, while logistic regression achieved an AUC of 0.82, indicating good-to-excellent model discrimination across all thresholds. These models consistently distinguished between responders and non-responders to priming on the basis of their biomechanical profiles

**FIG. 5:**
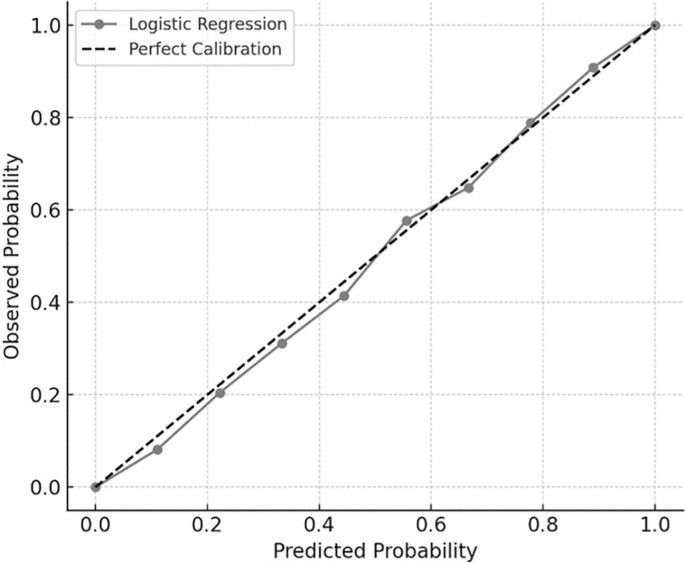
Calibration curve for logistic regression. Calibration plot comparing predicted and observed probabilities of RSI improvement (≥ 10%) across 10 risk deciles. The gray line with markers represents the logistic regression model, while the dashed diagonal line indicates perfect calibration. The close alignment between the two lines reflects strong agreement between predicted and actual outcomes.

To assess the reliability of the probabilistic predictions from the logistic regression model, a calibration curve was generated. As illustrated in Figure 5, predicted probabilities aligned closely with observed outcomes, with all deciles falling near the reference line. This indicates that the logistic model not only classified outcomes effectively but also produced well-calibrated probability estimates, thereby supporting its practical utility for individual-level performance forecasting.

## DISCUSSION

The present study examined the biomechanical and neuromuscular responses of elite youth female sprinters to three distinct sprintspecific priming strategies: resisted sprinting, overspeed sprinting, and technical wicket drills. Priming effects were assessed at two time points (24 h and 48 h) following a standardized baseline sprint condition. The findings indicate that both the type of priming activity and the post-intervention interval significantly modulated key performance indicators, including RSI, GCT, FT, and step length.

Among the priming activities, overspeed sprinting emerged as the most potent enhancer of key neuromuscular performance indicators, improving 50-m sprint speed and yielding the highest RSI values and the lowest GCT at 48 h post-activation. Despite differences in time frames, these findings align with the PAPE concept, which posits that assisted sprinting enhances motor unit recruitment, rapid force production, neuromuscular transmission, and contractile economy over an extended time course [2, 4, 20, 23]. Moreover, the positive changes observed in FT and RSI highlight the improved efficiency of the stretch–shortening cycle under this condition [30]. Mechanistically, these adaptations may reflect enhanced calcium sensitivity, increased rate of force development, and greater recruitment of type II motor units [6, 20, 23, 31].

Resisted sprinting, although less effective at elevating RSI, elicited meaningful improvements in step length and FT, particularly at 48 h post-activation.This suggests that horizontal force production and stride mechanics were positively influenced by increased posterior chain activation [9]. The delayed peak effects align with prior evidence indicating that resisted sprints elicit neuromechanical benefits through delayed potentiation rather than immediate excitation [20, 23]. In contrast, technical exercises such as wicket drills demonstrated the strongest influence on interlimb asymmetry and mediolateral sway, especially at 24 h post-intervention. These outcomes likely reflect the rhythmic, coordination-driven nature of wicket-based drills, which promote bilateral neuromuscular coordination and control [32, 33]. This observation is particularly relevant for youth athletes during key developmental stages, when motor reorganization and postural refinement are critical for sprint consistency and efficiency [27].

PCA divided the adaptation responses into two interpretable domains: force-time-related variables (PC1: RSI, GCT, and FT) and movement regulation (PC2: interlimb asymmetry, mediolateral sway, and step frequency). This result is consistent with previous literature emphasizing the dual imperative of power generation and motor control in sprinting [34]. Our k-means clustering identified a subgroup of “high responders,” characterized by pronounced gains in PC1 dimensions, predominantly following assisted sprinting and technical drills. These findings further support the notion that individual responses to priming activities are non-uniform and are likely influenced by baseline biomechanical factors, coordination efficiency (e.g., sprint technique), or intrinsic neuromuscular adaptability [2, 35]. Indeed, the predictive modeling analysis reinforced these patterns and underlying theoretical concepts. The priming strategy, baseline asymmetry, and step frequency emerged as the most relevant predictors of RSI improvement across all methods. Ensemblebased models (Random Forest, XGBoost) demonstrated robust discriminatory performance (AUC ≥ 0.83), while the logistic regression model showed high calibration accuracy. These results indicate that simple and practical biomechanical variables, when analyzed through multivariate methods, can effectively predict priming responsiveness and inform individualized intervention planning [36, 37].

From an applied perspective, tailoring priming selection to the intended adaptation window may enhance training planning [20]. For example, overspeed stimuli could be prioritized during taper phases where greater peak power output is required, while wicket drills may be more appropriate for sessions emphasizing rhythm and balance. The ability to rotate these training strategies according to athlete profile and training phase offers a flexible, evidence-based approach to designing effective priming sessions. Moreover, the identification of responder phenotypes may add an additional layer of nuance to warm-up programming. Biological factors such as ACTN3 and COL5A1 polymorphisms, muscle fiber composition, and mental readiness might influence priming effectiveness [38, 39]. Integrating genetic and sensor-derived data into decision-making systems could further improve the alignment of pre-activation strategies with individual profiles [38–40]. Finally, the growing availability of wearable systems capable of monitoring step frequency, interlimb asymmetry, and GCT in real time paves the way for dynamic optimization of priming interventions [40]. This trend reflects the broader shift toward individualized, adaptive training guided by predictive analytics.

Collectively, our findings indicate that RSI responses can be reliably predicted from a limited set of biomechanical variables, with priming activity exerting a predominant influence on performance indicators. The integration of PCA, clustering, and machine learning yielded robust insights into individual responsiveness, with overspeed and technical priming emerging as the most effective strategies for eliciting short-term neuromechanical adaptations. While this study provides compelling and novel evidence regarding the optimization of sprint performance, several limitations should be acknowledged. First, the sample consisted exclusively of elite youth female sprinters, which limits the generalizability of our findings to other populations. Second, the cross-sectional design precludes any conclusions about long-term adaptations. Third, the absence of external validation for the machine learning models warrants caution when extrapolating these results. Future research should employ longitudinal designs, incorporate electromyographic or electroencephalographic assessments, and evaluate model generalizability across sexes, age, and athletic disciplines. The inclusion of molecular markers, hormonal status, and fatigue-related indicators may further enhance our understanding of individual variability in response to distinct priming interventions across multiple time points (e.g., 6 to 48 h).

## CONCLUSIONS

The current study offers robust evidence that both the type of sprint priming strategy and the timing of post-activation assessments significantly influence neuromechanical and sprint-specific performance in elite youth female sprinters. Overspeed techniques produced the most consistent acute improvements in sprint performance, RSI, GCT, and FT; wicket drills enhanced interlimb asymmetry and coordination; and resisted sprinting improved stride mechanics. Multivariate analyses revealed two distinct domains of adaptation – force-time capacity and coordination – enabling the stratification of responder profiles. Machine learning models achieved strong predictive accuracy, with the type of priming strategy, baseline asymmetry, and step frequency emerging as key predictors of RSI improvement. In summary, these findings highlight the importance of individualized priming strategies grounded in biomechanical characteristics and endorse the use of integrated, data-driven approaches for sprint performance optimization and long-term athlete development.

## Practical Applications

Sprint priming strategies should be selected according to specific performance goals and the expected timing of neuromechanical responses. Overspeed techniques are most effective when applied 24–48 h before competition or high-intensity training, as they promote greater power output and enhance stretch–shortening cycle function. Technical drills are particularly useful for improving rhythm, interlimb asymmetry, and postural control, making them especially valuable for younger athletes or during recovery-oriented sessions. Resisted sprinting is best suited to strength-focused phases, contributing to longer strides and greater horizontal force production. Priming selection should be guided by individualized biomechanical assessments, with baseline asymmetry and step frequency serving as key predictors of responsiveness. To support this process, predictive models and machine learning techniques can offer valuable decision-making assistance when translated into user-friendly monitoring systems and implemented in collaboration with sport scientists and data analysts. This integration enables coaches to access tailored, evidence-based feedback without requiring specialized expertise in computational methods. Finally, wearable sensors and treadmill-based systems can facilitate the collection of relevant variables in both field and laboratory settings, allowing for more precise monitoring and individualized implementation of priming strategies within different training phases.
